# Structural and functional studies of STAT1 from Atlantic salmon (*Salmo salar*)

**DOI:** 10.1186/1471-2172-11-17

**Published:** 2010-03-30

**Authors:** Astrid Skjesol, Tom Hansen, Cheng-Yin Shi, Hanna L Thim, Jorunn B Jørgensen

**Affiliations:** 1 Norwegian College of Fishery Science, Faculty of Biosciences, Fisheries and Economics, University of Tromsø N- 9037 Tromsø, Norway; 2 Current address: Yellow Sea Fisheries Research Institute, Chinese Academy of Fishery Sciences, Qingdao 266071, PR China

## Abstract

**Background:**

Type I and type II interferons (IFNs) exert their effects mainly through the JAK/STAT pathway, which is presently best described in mammals. STAT1 is involved in signaling pathways induced by both types of IFNs. It has a domain-like structure including an amino-terminus that stabilizes interaction between STAT dimers in a promoter-binding situation, a coiled coil domain facilitating interactions to other proteins, a central DNA-binding domain, a SH2 domain responsible for dimerization of phosphorylated STATs and conserved phosphorylation sites within the carboxy terminus. The latter is also the transcriptional activation domain.

**Results:**

A salmon (*Salmo salar*) STAT1 homologue, named ssSTAT1a, has been identified and was shown to be ubiquitously expressed in various cells and tissues. The ssSTAT1a had a domain-like structure with functional motifs that are similar to higher vertebrates. Endogenous STAT1 was shown to be phosphorylated at tyrosine residues both in salmon leukocytes and in TO cells treated with recombinant type I and type II IFNs. Also ectopically expressed ssSTAT1 was phosphorylated in salmon cells upon *in vitro *stimulation by the IFNs, confirming that the cloned gene was recognized by upstream tyrosine kinases. Treatment with IFNs led to nuclear translocation of STAT1 within one hour. The ability of salmon STAT1 to dimerize was also shown.

**Conclusions:**

The structural and functional properties of salmon STAT1 resemble the properties of mammalian STAT1.

## Background

Interferons (IFNs) are cytokines that play a major role in host defense against viral pathogens [1,2]. Mammalian type I IFNs (IFNα/β) are produced by many cell types and confer antiviral activities on them, while type II IFN (IFNγ) is produced mainly by T lymphocytes and natural killer cells when stimulated by macrophage derived cytokines. IFNγ elicits broad effects, particularly on cells of the immune system. The transmission of both type I IFNs and IFNγ signals are dependent on the activation of the transcription factor STAT1 (signal transducer and activator of transcription). STAT family proteins are critical to the action of most cytokines and growth factors, as they are latent cytoplasmic transcription factors that directly activate signaling pathways upon being phosphorylated [3-5].

The activation of STAT is encompassed as part of evolutionary conserved pathways by which signals can be transduced from the membrane to the nucleus rapidly. The classical view is that type I IFN (IFNα/β) signals through STAT1/STAT2 heterodimers, while IFNγ signals through STAT1 homodimers [6,7]. The binding of secreted type I IFNs to the two subunit receptor (IFNAR1/IFNAR2) results in activation of the Janus-activated kinase 1 (JAK1) and tyrosine kinase 2 (TYK2), which are associated with the cytoplasmic tail of IFNAR1/2. The signal is cascaded further by tyrosine phosphorylation of STAT1 and STAT2 [3,8,9]. The STATs heterodimerize and together with interferon regulatory factor 9 (IRF9) form a complex named ISGF3. This complex enters the nucleus where it associates with specific promoter elements (termed the IFN-stimulated response element or ISRE) to activate the transcription of IFN-stimulated genes (ISGs) [9]. IFNγ signals through an IFNγ-specific receptor (IFNGR1/IFNGR2) to JAK1 and JAK2 resulting in tyrosine phosporylation and homodimerization of STAT1 [10]. STAT1 homodimers enter the nucleus and bind the IFNγ-activation site (GAS) which is present in the promoter of certain ISGs [3,11]. However, in addition to the phosphotyrosine SH2 domain interactions of the active forms of STATs, unphosphorylated STATs can form dimers of a different conformation through their N-terminal domain [12]. Also, STAT1 can be found in both the cytoplasm and the nucleus without cytokine stimulation of cells [13].

Facilitated nuclear translocation of such large complexes requires the nuclear pore complex [5,14]. STAT1 and STAT2 do not contain classical nuclear localization signals (NLS) which is normally necessary to be recognized by the importin receptor, but dimerization of STATs results in conformational changes that establish NLS activity [13,15,16]. After activation of their target genes, STATs are dephosphorylated, released from the DNA and shuttled back to the cytoplasm [12,17,18]. Consistent with the importance of this pathway in mediating the actions of IFNs, mice with no STAT1 have no innate response to either bacterial or viral infections as a result of dysfunctional IFN signaling [19]. Moreover, a number of viruses have the capacity to block the activation of STAT1 by IFN to evade the defense from the host immune system [20].

Recently, significant progress has been made in identifying and characterizing fish genes related to the IFN system, including several type I IFN genes [21-23], IFNγ [24-28] and antiviral genes [23]. Far less is known about the factors that are involved in IFN-signaling in fish, including the JAK-STAT pathway, although STAT1 homologs have been cloned from several fish species [29-31]. Additionally, a STAT2 gene was recently identified in salmon [32], and TYK2 and JAK1 have been cloned from green pufferfish (*Tetraodon fluviatilis*) [33,34].

In the present work we describe the identification and characterization of a STAT1 gene from Atlantic salmon. To get insight into the role of STAT1 in response to cytokines and viruses in salmon we have studied the expression and activation of STAT1 in primary leukocyte cultures and in different salmonid cell-lines upon type I and type II IFN treatment and viral infections. The ability of STAT1 to be phosphorylated and to translocate to the nucleus is critical for its role as a transcription factor. By employing a salmon STAT1 antibody the localization of STAT1 in different cells in response to IFN-treatment were studied. Furthermore, STAT1 phosphorylation was detected using a phosphotyrosine specific antibody after treatment with the same stimulants. Such studies have not been performed in any teleost species earlier. Both IFN-a1 and IFNγ treatment led to tyrosine phosphorylation and STAT1 was also shown to be translocated to the nucleus after stimulation with IFN-a1 and IFNγ. We also show, using two different *in vitro *methods, that salmon STAT1 is able to form dimers.

## Results

### Cloning of STAT1

**Figure 1:**
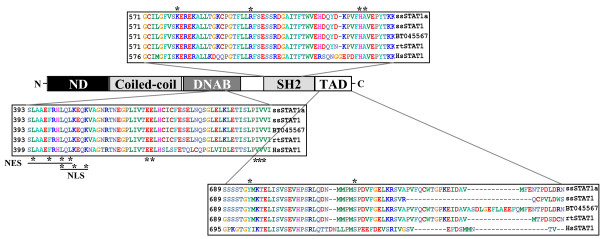
**Salmon STAT1 protein harbors conserved domains and sequences**. The NCBI conserved domains database and ClustalW alignment was combined to depict the schematic presentation of STAT1. *Abbreviations: *ND = amino-terminal domain, DNAD = DNA-binding domain, SH2 = Src Homology 2 domain, TAD = transcriptional activation domain, NES = nuclear export signal, NLS = nuclear localization signal, ss = Atlantic salmon, rt = rainbow trout, hs = human. Asterisks indicate conserved residues with importance for the functional activity within the domains.

With primers derived from a rainbow trout (*Oncorhynchus mykiss*) STAT1 sequence we obtained a 2.3 kB DNA fragment by PCR using cDNA from salmon ovaries and HK. Sequencing of the cloned fragment revealed a 2 274 base pairs long open reading frame which translated into an amino acid sequence with strong homology to the rainbow trout STAT1 sequence (97.6% identity, 98.8% similarity). This salmon sequence was 3 amino acids longer than the rainbow trout sequence, and had a predicted molecular mass of 87.5 kDa. While preparing this manuscript two other STAT1 sequences from Atlantic salmon were submitted to the GenBank database (accession numbers EU016199[31] and BT045567). These two sequences shared 96.3% and 98.3% identity, respectively, to our clone. Our sequence was named ssSTAT1a (GenBank accession number GQ325309). The major differences found between the three salmon sequences were located in the C-terminal end (Figure 1), where EU016199 has two single nucleotide deletions when compared to ssSTAT1a (T missing in position 2190, and T missing in position 2201), which leads to frame shifts and subsequently a premature stop. In BT045567 is found an insertion of 39 nucleotides (13 amino acids) in the C-terminus when compared to ssSTAT1a (Figure 1). PCR primers were designed in an attempt to pick up, and to distinguish between the ssSTAT1/EU016199 and BT045567 variants. The primer pair (rtSTAT1fw + ssSTATgap rev, Table 1) would detect only the BT045567 variant and give a PCR product of 72 nucleotides. However, using this strategy we were unable to amplify the BT045567 variant in cDNA from both stimulated and unstimulated salmon leukocytes (results not shown).

**Table 1:** Primer sequences and their applications in this study.

Primer name	Sequence 5' - 3'	Application
rtSTAT1fw	CACCATGGCCCAGTGGTGCCAGCTGCA	Gene cloning/sequencing
rtSTAT1rev	CTACTATCAGTTGCAGTCCGAGTCAGGTG	Gene cloning/sequencing
ssSTAT1 2204 fw	AGTGTTGGACTGGTCCTAAGGA	Isoform detection (PCR)
ssSTAT 1gap rev	TGAAATTCTTCAGCTAAAAACTCTC	Isoform detection (PCR)
ssSTAT1fw	CGGGCCCTGTCACTGTTC	qPCR
ssSTAT1rev	GGCATACAGGGCTGTCTCT	qPCR

ssSTAT1 probe	ACCACCAAGGAATGTTC	qPCR

**Figure 2:**
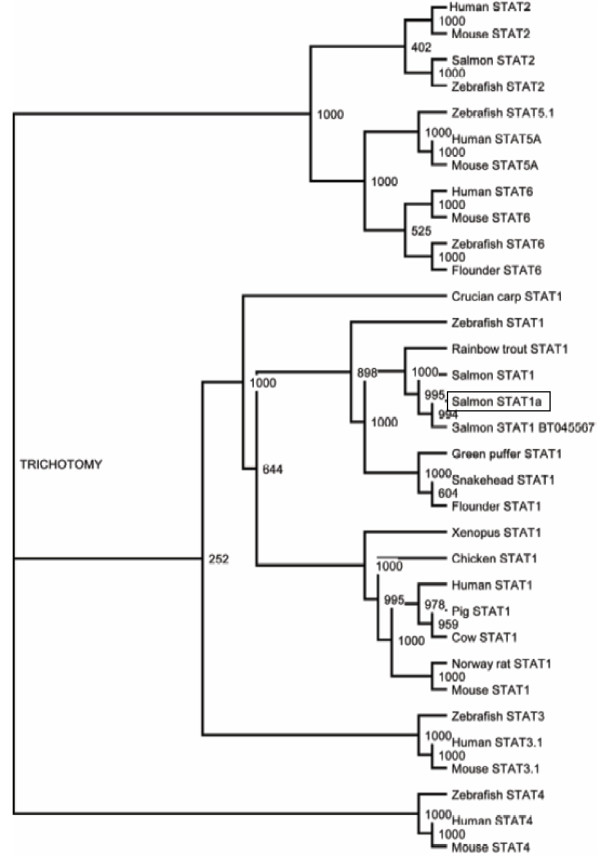
**STAT1 phylogeny**. An un-rooted phylogenetic tree of STAT1 and other STAT proteins based on sequences aligned by ClustalW was constructed using the neighbor-joining algorithm. Bootstrap value = 1000.

The amino acid sequence of ssSTAT1a was compared to known STAT1 sequences from other species (Additional file 1, Supplemental Table S1) and revealed high homology, also with human and rat STAT1 (>60% identity, >80% similarity). The non-salmonid teleost species that was found to share the highest homology to salmon STAT1 was snakehead (*Channa argus*) STAT1 (80.1% identity, 90.4% similarity), whereas crucian carp (*Carassius auratus*) had the lowest identity of the compared species (58.4% identity, 75.7% similarity). In the presented phylogenetic tree ssSTAT1a is located in the same clade as other piscine STAT1 sequences when high bootstrap values were applied (Figure 2).

The amino acid sequence of the ssSTAT1a clone contained several prototypic features and conserved domains crucial for STAT1 functions (Figure 1). In the most C-terminal part of the sequence, the transcriptional activation domain (TAD), a conserved serine phosphorylation site was found in position 719 (homologous to serine 727 in human STAT1). This phosphorylation site is known to be crucial for the activation of transcription [35]. A conserved tyrosine residue was located in position 695 (701 in human). Phosphorylation of this tyrosine residue is crucial for activating the STAT molecule and thereby enabling it to interact with the phosphotyrosine binding pocket located in the Src homology 2 (SH2) domain of other STAT molecules [36]. An analysis of this conserved domain (using NCBI CDD [37]) revealed that a phosphotyrosine binding pocket was located in SH2 of ssSTAT1a, where all four of the residues composing this feature was present, which of the conserved arginine at position 597 is believed to be the most crucial for forming H-bonds with phosphate oxygens of the phosphotyrosine side chain [38]. In addition residues important for DNA-binding (DNAB), nuclear import (NLS) and export (NES) [15,39-41] were found to be conserved between mammalian and teleost STAT1. Residues crucial for these functions are marked with asterisks in Figure 1.

### Tissue distribution of STAT1 mRNA

**Figure 3:**
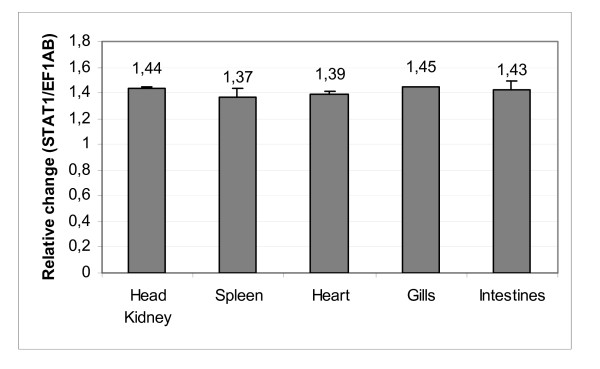
**STAT1 mRNA is evenly distributed in salmon tissues**. STAT1 mRNA levels in the salmon tissues head kidney, spleen, heart, gills and intestines was detected by qPCR analysis. The expression levels are presented as change in expression relative to EF1AB. Samples from 10 fish were tested.

Quantitative real-time PCR (qPCR) was undertaken to examine expression of STAT1 mRNA in different salmon tissues. A uniform distribution of ssSTAT1a mRNA was observed in all tissues tested, including head kidney, spleen, heart, gills and intestines, derived from 10 unvaccinated and healthy salmon controls (Figure 3).

### Induction and expression of STAT1 protein in salmon primary cells

**Figure 4:**
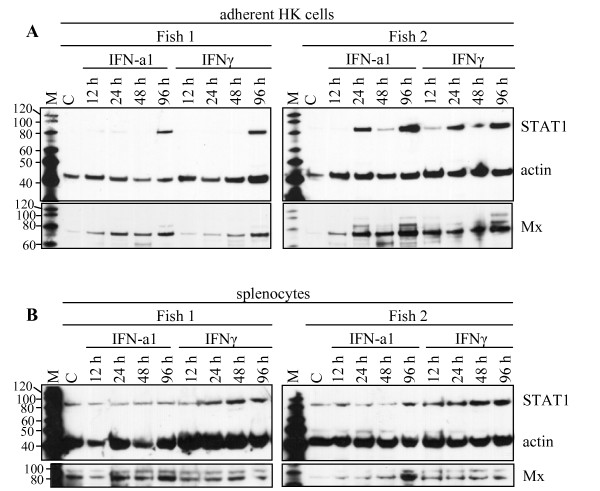
**SDS-PAGE followed by Western blot showing expression of STAT1 protein in (A) adherent head kidney leukocytes and (B) splenocytes from two individuals upon stimulation with IFN**. The samples were harvested 12, 24, 48, and 96 h after stimulation with IFN-a1 (10 U/mL) and IFNγ (200 ng/mL). The unstimulated control, C, was harvested at the 48 h time-point. STAT1 protein was detected simultaneously with actin which was used as a loading control. The membranes were stripped and reprobed with an anti-Mx-antibody as a control for the IFN-activity. M = MagicMark molecular weight marker.

For detection of STAT1 protein in salmon cells, a custom made peptide antibody was applied for Western blotting. Primary cells from salmon were subjected to Western blotting upon stimulation with both type I and type II IFN (Figure 4). In unstimulated adherent HK leukocytes (mostly monocytes and macrophages) (Figure 4A) the STAT1 protein was barely detectable, while the STAT1 levels were increased upon both IFN-a1 and IFNγ stimulation, showing maximum levels at 96 h post stimulation. Individual variations among the fish tested were observed, as exemplified in Figure 4, where increased STAT1 levels upon stimulation were induced earlier in Fish 2 compared to Fish 1. In splenocytes (mostly lymphocytes) (Figure 4B) a more even STAT1 expression pattern was seen when comparing non-treated cells with the stimulated cells, indicating that STAT1 expression levels in splenocytes were less affected by IFN treatment, but with a slight up-regulation after IFNγ stimulation. Mx protein was detected on the same membranes after stripping and reprobing with an Mx antibody. For HK leukocytes the relative increase in Mx expression occurred earlier than the increase in STAT1 expression, while the splenocytes showed a constitutive Mx expression which paralleled the STAT1 expression.

### Transcription levels of salmon STAT1 mRNA in salmon HK leukocytes

**Figure 5:**
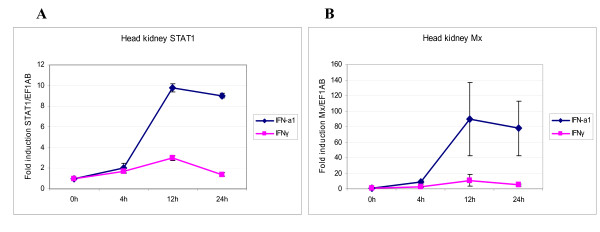
**QPCR showing expression of STAT1 and Mx mRNA in IFN-stimulated adherent head kidney leukocytes**. The cells were stimulated with 10 U/mL of recombinant Atlantic salmon IFN-a1 or 200 ng/mL of recombinant rainbow trout IFNγ. The mRNA levels were normalized against EF1AB. (A) STAT1 mRNA expression after 4, 12 and 24 h of stimulation. (B) Mx mRNA expression after 4, 12 and 24 h of stimulation. The results are an average of samples from three fish and presented as fold increase relative to unstimulated head kidney cells. Experiments were performed twice with reproducible results. Error bars show standard deviation.

To determine whether the increase in STAT1 protein expression upon type I and type II IFN treatment of HK leukocytes were induced at transcriptional levels, the amount of mRNA was measured at various times after stimulation, by real-time RT-PCR. A representative experiment is shown in Figure 5. Both types of IFNs induced STAT1 expression levels slightly above unstimulated cells. In cells isolated from three fish, IFN-a1 caused an average increase in transcription of STAT1 ranging from a 2-fold induction at 4 h through nearly 10-fold induction at 12 h and then a small decline at 24 h (9-fold induction). The levels of STAT1 transcripts after stimulation with IFNγ did barely change relative to the unstimulated control at the time points investigated, with a peak at 3-fold increase at 12 h (Figure 5A). To verify the activity of the IFNs used, the levels of Mx transcripts were recorded in the same samples. At 24 h the Mx transcripts were induced to a level 90 times above the unstimulated cells when IFN-a1 was added to the cells, whereas the IFNγ-induced transcription was 11-fold increased at the same time point (Figure 5B).

### STAT1 protein expression levels in TO and CHSE-214 cells upon IFN-treatment and viral infections

**Figure 6:**
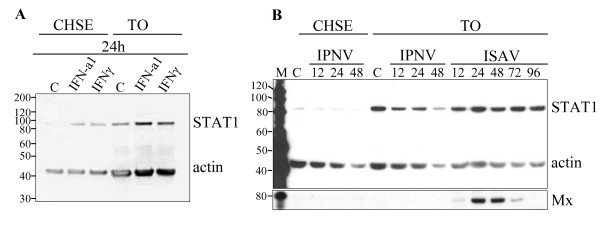
**SDS-PAGE followed by Western blot showing expression of STAT1 protein in salmonid cell-lines**. Antibodies against salmon STAT1 (α-STAT1 1:2,000), actin (α-actin, 1:1,000) and Mx (α-Mx, 1:1,000) were used. (A) CHSE-214 and TO cells were harvested 24 h after stimulation with IFN-a1 (10 U/mL) and IFNγ (200 ng/mL) along with an unstimulated control, C. (B) CHSE-214 and TO cells were infected with IPNV (MOI = 4) and harvested at 12, 24 and 48 h p.i. TO cells were also infected with ISAV (MOI = 4) and harvested at 12, 24, 48, 72 and 96 h p.i. Actin was used as a loading control and Mx protein detected after the membrane was stripped and reprobed. M = MagicMark molecular weight marker.

Two salmonid cell-lines, TO and CHSE-214 cells, known to be permissive for several viruses, were tested for expression of STAT1 protein upon stimulation with different cytokines or virus. In unstimulated CHSE-214 cells expression of STAT1 protein was barely detectable, while a modest induction upon IFN-a1 or IFNγ stimulation was found (Figure 6A). Infection with IPNV at MOI of 4 for 12, 24 or 48 h, did not increase protein expression above the level of uninfected cells (Figure 6B). Also infections with Norwegian field isolates of highly virulent IPNV strains showed the same results (results not shown). Also induced STAT1 levels were found in TO cells after 24 h of exposure to IFN-a1 or IFNγ. However, neither infection with IPNV (for 12, 24 and 48 h) nor ISAV (for 12, 24, 48, 72 and 96 h) increased STAT1 expression. Mx protein was, however, induced after 24 h and 48 h of ISAV infection.

### Subcellular localization of STAT1

**Figure 7:**
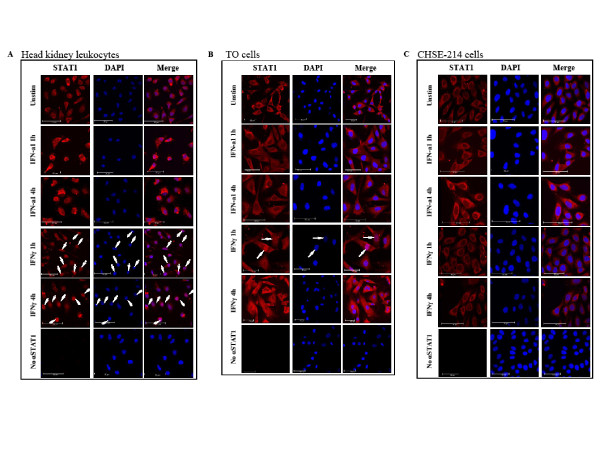
**Subcellular localization of STAT1 in different cell-types after stimulation with IFN-a1 and IFNγ**. The cells were treated with IFN-a1 (10 U/mL) or IFNγ (200 ng/mL) for 1 or 4 h or left unstimulated before fixed in 4% paraformaldehyde and stained for STAT1 (red). Nuclei were stained with DAPI (blue). (A) Salmon adherent head kidney leukocytes. Translocation of STAT1 to the nucleus took place at 1 h and at 4 h after stimulation with IFNγ as indicated by arrows. (B) TO cells. Translocation of STAT1 to the nucleus took place about 1 h after stimulation with IFNγ as indicated by arrows. (C) CHSE-214 cells. No translocation of STAT1 to the nucleus was observed. A control with no α-STAT1 is included for each of the cell types.

**Figure 8:**
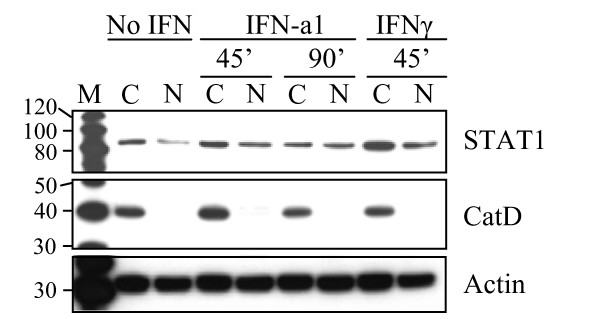
**Nuclear localization of STAT1 in TO cells after stimulation with IFN-a1 and IFNγ**. The cells were treated with IFN-a1 (10 U/mL) for 45 or 90 min or IFNγ (200 ng/mL) for 45 min or left unstimulated. Nuclear extracts (N) and cytoplasmic fractions (C) were separated using NE-PER Nuclear and Cytoplasmic Extraction Reagents (Thermo scientific). The extracts were subjected to SDS-PAGE and Western blotting. STAT1 was detected in both N and C fractions, but lesser STAT1 was observed in the nuclear fraction of the unstimulated cells. An antibody directed against a strictly lysosomal protein, Cathepsin D (CatD) was used as a control for cytoplasmic localization. After stripping the membrane, an actin antibody was applied as a loading control. M = MagicMark molecular weight marker.

The subcellular localization of STAT1 after type I and II IFN stimulation in salmon primary leukocytes and salmonid cell-lines was examined by confocal laser scanning microscopy. For this purpose cells were seeded on coverslips and stimulated for 1 h and 4 h with 10 U/ml IFN-a1 and 200 ng/ml of IFNγ. The cells were then fixed and immunostained for STAT1 as described in the Methods section. In all cell types studied, STAT1 (red dye) localized exclusively to the cytoplasm in unstimulated cells (Figure 7A, B and 7C). In primary cells from Atlantic salmon HK, a relocalization of STAT1 from the cytoplasm to the nucleus took place after stimulation with IFNγ for 1 h and STAT1 was still retained in the nuclei at 4 h (Figure 7A), although individual variations in the kinetics occurred among the 3 fish studied. In TO cells the response to IFNγ seemed more transient than in the primary cells. At 1 h after IFNγ stimulation, some of the nuclei in the TO cells had already been stained with STAT1 and after 4 h STAT1 had relocated to the cytoplasm (Figure 7B). CHSE-214 cells did not respond to IFNγ with STAT1 translocation at the time points studied (Figure 7C). Relocalization of STAT1 after IFN-a1 stimulation was not observed by microscopy in any of the cell types at the time points chosen in this study. As a consequence of the inability of detecting STAT1 in the nucleus by immunostaining and confocal laser scanning microscopy following IFN-a1 stimulation, nuclear extracts of IFN-a1 stimulated TO cells were examined for the presence of STAT1 (Figure 8). STAT1 was detected in both cytoplasmic and nuclear extracts from cells stimulated with IFN-a1 for 45 or 90 min or IFNγ for 45 min, but also, to a lesser extent, in unstimulated cells. As a control for the separation of the cytoplasmic and nuclear fractions, an antibody directed against a strictly lysosomal protein, Cathepsin D (CatD) was used as a control for cytoplasmic localization [42].

### Salmon STAT1 is phosphorylated upon IFN treatment

**Figure 9:**
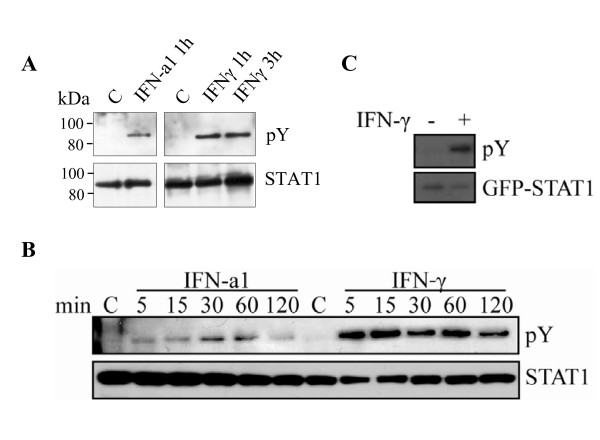
**STAT1 is phosphorylated in response to IFN-a1 and IFNγ**. Cells were either treated with IFN-a1 (10 U/ml) or IFN-γ (200 ng/ml) or left untreated. Cells were harvested at indicated time points and endogenous STAT1 were immunoprecipitated with α-STAT1 from the whole cell extracts. Tyrosine phosphorylated STAT1 was detected by immunoblotting using anti-phosphotyrosine antibody (pY, upper panel). The total amount of immunoprecipitated STAT1 was detected with α-STAT1 antibody (lower panel). (A) Head kidney leukocytes. (B) TO cells. (C) TO cells were transfected with a GFP-ssSTAT1a construct. After 48 h, the cells were treated with IFNγ (200 ng/ml) for 30 min, or left untreated. Cells were lysed and GFP-tagged proteins were immunoprecipitated with α-GFP. Phosphorylated GFP-STAT1 was detected by immunoblotting using anti-phosphotyrosine antibody (upper panel). The total amount of immunoprecipitated GFP-STAT1 was detected with α-GFP antibody (lower panel).

Tyrosine phosphorylation is a key step in STAT1 mediated IFN-signal transduction. To address whether the conserved tyrosine residue found in the salmon STAT1 sequence is phosphorylated upon stimulation, an antibody specific to phosphorylated tyrosine was used. IFNγ-stimulated primary leukocytes were harvested and the STAT1 antibody applied in order to immunoprecipitate STAT1 molecules. The pulled-down material was subjected to SDS-PAGE and Western blotting, and the membrane incubated with a tyrosine phospho-specific antibody. By IFNγ-stimulation phosphorylated STAT1 was detected in adherent HK cells and splenocytes after both 1 h and 3 h (Figure 9A), although cells from different individuals responded differently to this treatment (results not shown). Similarly, STAT1 was phosphorylated upon stimulation with IFN-a1 at the same time-points (Figure 9A). Phosphorylation of STAT1 was also confirmed in TO cells (Figure 9B). A time-course study in these cells showed that with IFNγ treatment STAT1 was phosphorylated already after 5 min and peaked at 15 min, while in IFN-a1 treated cells a weak band was detected at 5 min, increased at 15 min and then remained relatively constant up to 60 min. After 120 min phosphorylation was regressing for both types of IFN. Phosphorylated ectopically expressed ssSTAT1a was detected after 30 min by the tyrosine phospho-specific antibody (Figure 9C). This experiment confirms that the cloned ssSTAT1a can be tyrosine phosphorylated upon IFN treatment, which is an important characteristic of this protein.

### Salmon STAT1 homodimerisation

STAT1 is known to form homodimers upon phosporylation [10]. However, dimerization of unphosphorylated STAT1 in the cytoplasm is also reported [43] By employing the yeast two-hybrid (Y2H) system we were able to demonstrate a strong salmon STAT1-STAT1 interaction (Table 2). Interacting proteins were assessed by the ability of growth on complete medium deficient in histidine (TDO) or histidine and adenine (QDO). Growth was recorded at day 4, when the positive control expressing p53 fused to Gal4_DBD _and SV40 T-antigen fused to Gal4_AD _showed massive growth on QDO (++++). The specificity of the interaction was tested with negative controls, and confirmed negative (no growth on TDO nor QDO) by co-expressing the Gal4_AD_-ssSTAT1a fusion protein both with Gal4_DBD _alone and with Gal4_DBD _fused to the human LaminC protein, which is known to be a non-interacting protein [44,45].

**Table 2:** Interaction between ssSTAT1a and ssSTAT1a detected in the yeast two-hybrid system.

**Gal4_DBD_-fusion**	**Gal4_AD_-fusion**		
	
	**ssSTAT1a**	**SV40 T-antigen**	**No insert**
	
ssSTAT1a	++++ (TDO) +++ (QDO)	ND	-
P53	ND	++++ (TDO) ++++ (QDO)	-
LamC	-	-	-
No insert	-	-	-

++++ strong interaction, - no interaction, QDO quadruple drop out medium, TDO triple dropout medium, ND not determined.

**Figure 10:**
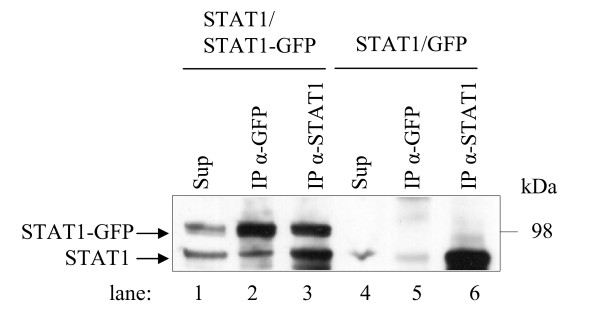
**Co-IP analyses of the ssSTAT1a - ssSTAT1a interaction**. ssSTAT1a was co-expressed with GFP-ssSTAT1a or pEXP-GFP (negative control) in HEK-293 cells and the lysed cells subjected to IP with a STAT1 antibody (α-STAT1) or a GFP antibody (α-GFP). Samples were analyzed along with the total cell lysate (sup) by SDS-PAGE followed by Western blot using α-STAT1.

A co-IP analysis of over-expressed ssSTAT1a and ssSTAT1a-GFP fusion constructs in HEK-293 cells verified the ssSTAT1a-ssSTAT1a interaction further. The Western blot in Figure 10 was probed with α-STAT1 and showed expression of both ssSTAT1a and ssSTAT1a-GFP in the lysate (lane 1, ~ 85 kDa and ~ 110 kDa respectively). ssSTAT1a was co-precipitated along with ssSTAT1a-GFP when the GFP antibody was applied for the IP (lane 2) and α-STAT1 precipitated both ssSTAT1a and ssSTAT1a-GFP (lane 3). As a negative control ssSTAT1a was co-transfected with the pEXP-GFP vector. The GFP antibody did not precipitate ssSTAT1a (lane 5) whereas α-STAT1 did (lane 6).

## Discussion

IFN induced immune responses in which STATs are required are among the best understood signaling systems in mammals. Although a number of proteins involved in the JAK/STAT signaling pathway have been cloned from fish, less is known about their function and whether the signaling resembles mammalian systems.

We have here cloned a cDNA that corresponds to the salmon STAT1 gene. A clustalW alignment confirmed that the cloned sequence was a STAT1 homolog sharing extensive amino acid identity with other salmon STAT1 isoforms and trout STAT1 (Table 1 and Figure 1). The 2 274 nucleotides open reading frame of the cloned cDNA has a structural arrangement of functional motifs that is similar to mammalian STAT1 suggesting that the salmon cDNA encode a functional protein.

So far STAT1 has been found in multiple fish species, including pufferfish, zebrafish (*Danio rerio*), rainbow trout, Atlantic salmon and Japanese flounder (*Paralichthys olivaceus*). Previous expression data, along with data presented here has revealed that piscine STAT1 is widely expressed in many tissues [30,31,46-49]. However, data concerning functional activity in lower vertebrates, such as STAT1 phosporylation and cellular localization upon stimulation of cells, is scarce. The presented data demonstrates for the first time that a teleost STAT1 protein is being activated by IFNs. Salmon STAT1 was shown to be tyrosine phosphorylated upon IFN-a1 and IFNγ stimulation of leukocytes, and additionally in TO cells. Also, relocalization of STAT1 into the nucleus of leukocytes and TO cells was observed following IFNγ stimulation. Our data show a more evident response for IFNγ than for IFN-a1 when studying nuclear translocation of STAT1 by microscopy. This was also consistent with the levels of phosphorylated STAT1 observed upon the different IFN-stimulations where IFNγ consistently gave higher levels compared to IFN-a1. STAT1 is believed to be involved in both type I and type II IFN signaling and their distinct responses could be due to unequal concentrations or activity of the cytokines used, or be dependent on differences in the kinetics of forming the complexes that enter the nuclei. We also showed that ssSTAT1a is able to form homodimers which is thought to be a prerequisite for entering the nucleus due to lack of a functional nuclear localization signal in its monomeric form [16]. In unstimulated cells STAT proteins can exist as stable unphosphorylated dimers or monomers which are also shuttled over the nuclear membrane [50-52] and are able to regulate gene expression in unconventional manners [5], consistent with the observed presence of STAT1 in nuclear extracts of unstimulated cells.

The TO cell line is derived from salmon HK and it consists of heterogeneous cell types [53]. The CHSE-214 cells are embryo cells derived from Chinook salmon (*Oncorhynchus tshawytscha*) [54]. Both these cell lines are widely used as experimental systems to study immune responses in salmon [47,55-60]. According to our results, the expression of STAT1 protein seems to be up-regulated upon type I and type II IFN treatment in both the cell lines and also in primary HK leukocytes. While the presented Western data are not quantitative, we provide qPCR data showing that type I IFN induce STAT1 expression in HK leukocytes to a greater extent (10-fold) than type II IFN (3-fold), and also by different kinetics, where the response to type II IFN peaked at an earlier time-point. Unlike the HK leukocytes, the splenocytes, which are mostly lymphoid-like cells, did not show increased abundance of STAT1 upon IFN-stimulation. The up-regulation of salmon STAT1 transcripts after IFN-a1 stimulation of TO cells is reported by others [31,47], additionally the type I IFN inducer poly (I:C) has been shown to induce STAT1 mRNA expression in RTG-cells [48].

Following encounter with viral pathogens, CHSE-214 and TO cells did not seem to boost the levels of STAT1 protein, but stayed relatively constant. Mx-protein was induced 24-48 h after infection with ISAV, but did not respond to IPNV infection, which is in compliance with results reported earlier [57,61,62]. The uniform expression patterns in salmon tissues and in different cell-types treated in various ways indicate that it is likely that STATs are present in the cytoplasm in most resting tissues, alert and ready to be activated upon cellular receptor signaling. The signal transduction and activation of ISGs may lead to a feedback loop that amplifies IFN-responses and induces STAT1 in a secondary manner, STAT1 itself being an ISG. This is shown in human cells-lines where IFNγ-induced IRF-1 in concert with CREB binding protein acts as key up-regulator of STAT1 mRNA transcription by binding to a combined IRF-E/GAS element in the STAT1 promoter [63]. Mutual regulation of STAT1 and IRF-1 indicates an intracellular amplifying circuit in response to IFN.

The STAT1 complexes formed in response to type I IFN in salmonid cells are presumably, like in mammals, distinct from those formed in response to type II IFN. Masking of the epitope for the STAT1 antibody after responding to IFN1a is a plausible explanation to the inability to detect nuclear STAT1 by confocal microscopy examination upon IFN-a1 stimulation. Under the denaturing conditions of SDS-PAGE and Western blotting STAT1 was detected in the nucleus of TO cells after treatment with IFN-a1. Also a small portion of STAT1 was detected in nuclear extracts of unstimulated cells, which is consistent with observations of STAT1 as a constitutive transcriptional regulator in mammalian systems [13,64,65]. Unlike TO cells and primary leukocytes, no relocalization of STAT1 from the cytoplasm to the nucleus was found upon IFNγ treatment of CHSE-214 cells. This could be due to the apparent low levels of endogenously expressed STAT1 protein in these cells. However, the expression levels were evaluated by Western blotting and the low detectable levels may be due to a lower affinity of the STAT1 antibody to Chinook salmon STAT1. Nevertheless, the levels of STAT1 in CHSE-214 were induced by both types of IFN, but considerably later (24 h, Figure 6) than the time points used for the confocal microscopy examination. No nuclear extracts were made in order to examine STAT1 expression in these cells. The presence of the IFN receptors (IFNAR and IFNGR) in the different cell-lines is also uncertain although a putative IFNγ-receptor was recently identified in rainbow trout [66]. The differences in response endorse the assumption that there are distinct proteins involved in signaling from type I and type II IFNs. The presence of a STAT2 gene in salmon was recently reported [32], and the first teleost importin alpha gene was recently cloned from red seabream (*Pagrus major*) [67], both findings adding to the assumption that IFN signaling in fish resembles that of mammals.

Unlike the (most common) action of human IFNγ [24,68], IFNγ in salmonids up-regulates Mx expression [55,69]. This might be a consequence of indirect stimulation, as fish IFNγ can activate type I IFN [69] (and own unpublished data). Interestingly, recombinant IFNγ activates an ISRE-containing reporter-construct in a dose dependent manner whereas constructs containing only GAS elements give no response to either type of IFNs as shown by Castro et al. [70]. This finding suggests that cross-talk between IFN signaling pathways occurs in fish. Overlapping effects of type I and II IFNs are also described in mammals [71,72] In the absence of exogenously administered IFNs, over-expression of ssSTAT1a by transfection in TO cells activated the Mx-promoter at a very moderate level (Additional file 2, Supplemental Figure S1). Furthermore over-expression of ssSTAT1a gave no additionally increased Mx-promoter activity upon type I or II IFN supplement. This is probably due to lack of stoichiometric balance of factors that activate, or interact with, the over-expressed STAT1 molecules. Similar results are observed in mammalian cells with a slightly different IFNγ-responsive reporter [13].

Naturally occurring truncated forms of STATs can act as competitors of functional STATs and inhibit transcriptional activation [73]. The ssSTAT1a gene differs from two other salmon STAT1s published in GenBank. The presence of more than one STAT1 isotype in salmon implies distinct functions for the different STAT1s, possibly at the level of transcription activation as the main differences are located in the TAD. The presented ssSTAT1a is longer than EU016199 (ssSTAT1) [31] while shorter than BT045567. Our attempt to detect both ssSTAT1a and BT045567 transcripts in salmon primary cells failed, and only ssSTAT1a was detected independent of the type of treatment the cells were subjected to. Other cell-types, signals or cellular conditions might favor BT045567 expression.

## Conclusions

We have cloned a salmon STAT1 isoform possessing conserved structural domains. Expressional and functional studies of this gene suggest that salmon use the specialized JAK/STAT pathway for cytokine signaling as STAT1 is tyrosine phosphorylated and translocates to the nucleus upon IFN stimulation. Further details related to JAK/STAT signaling and viral infections of this economically important fish needs to be elucidated. The ability of IPNV to impair STAT1 activation/phosphorylation is currently being examined, and the interacting ability of activated STAT1 needs to be addressed.

## Methods

### Fish

For *in vitro *cell-culture studies non-vaccinated Atlantic salmon, *Salmo salar *L., strain Aquagen standard (Aquagen, Kyrksæterøra, Norway), 500-1000 g, was obtained from Tromsø Aquaculture Research Station (Tromsø, Norway). The fish were kept at 6 to 12°C in tanks supplied with running filtered sea water, and were fed according to appetite on commercial, dry food.

For tissues expression studies, non-vaccinated Atlantic salmon (~30 g), were obtained from SalmoBreed (Norway). The fish were kept in 150 l fresh water at 10 to 13°C, with an oxygen saturation >65% and were fed according to appetite on commercial, dry food. The fish were negative for the presence of infectious pancreatic necrosis virus and salmon alphavirus when screened by qPCR prior to the experiment.

The experiment was approved by the National Committee of Ethics as required by Norwegian law. Proper anaesthetics have been used and the number of fish was kept as low as possible to still get statistically significant results.

### Cell cultures and virus

Atlantic salmon head kidney (HK) or spleen leukocytes were isolated as described by Jørgensen et al. [74]. The density of the leukocyte suspensions was adjusted to 7 × 10^6 ^cells/ml. One ml of HK leukocytes was plated per well in 24-well plates in L-15 medium with 5% FBS, whereas one ml splenocytes were plated in RPMI-1640 with 5% FBS. After approximately 24 h of incubation at 14°C, the cells were washed with culture medium prior to stimulation.

Chinook salmon embryo cells (CHSE-214) [54] were grown as monolayers at 20°C, 5.0% CO_2 _in Eagle minimal essential medium with GlutaMAX (EMEM+GlutaMAX, Invitrogen) supplemented with 100 μg/ml streptomycin, 60 μg/ml penicillin, 1% non essential amino acids and 8% fetal bovine serum (FBS, Euroclone). For infection experiments and Western analyses CHSE-214 cells were seeded into 24-well plates (2 × 10^5^cells/well) and grown to 80% confluence prior to infection.

TO cells originating from Atlantic salmon head kidney [53] were grown as monolayers at 20°C, 5.0% CO_2 _in Eagle minimal essential medium with GlutaMAX (EMEM+GlutaMAX, Invitrogen) supplemented with 100 μg/ml streptomycin, 60 μg/ml penicillin, 1% non essential amino acids and 5% fetal bovine serum (FBS, Euroclone).

HEK-293 cells (GP-293; Clontech) were maintained at 37°C, 5.0% CO_2 _in EMEM supplemented with 100 μg/ml streptomycin, 60 μg/ml penicillin, 4 mM L-glutamine and 10% fetal bovine serum (FBS).

Infectious pancreatic necrosis virus (IPNV) of the N1 strain, serotype Sp, was used in this study. The experiments were performed with a multiplicity of infection (MOI) of 4 infectious particles in CHSE-214 or TO cells. After absorption of the virus for 3-4 h in serum free culture medium, the medium containing virus was carefully removed from the cells. The infection was then carried out at 17.5°C in the presence of 2% FBS and cells harvested at different time points. Propagation and titration of virus was performed as described in Pedersen et al [75].

Infectious salmon anemia virus (ISAV) of the Norwegian reference strain Glesvaer 2/90 [76] (isolate ISAV4, hemagglutinin GenBank accession number AF220607) was kindly provided by Dr. B. Annexing, National veterinary institute, Oslo, Norway, and used to infect TO cells at a MOI of 4. The infection was carried out at 17.5°C in the presence of EMEM with 2% FBS and the cells harvested at different time points.

### Stimulation of cells

Recombinant Atlantic salmon IFN-a1 (previously named IFN-α1) was produced in HEK293 cells as described elsewhere [77]. The salmon IFN-a1 used in this study had a titer of 24 237 U/ml as estimated by the formula given by Renault et al.[78]. IFN-a1 was administered to the cells at a concentration of 10 U/mol in EMEM containing 2% FBS. Two hundred Ngami of recombinant rainbow trout IFNγ [24] were used for stimulation of the cells.

### Cloning of STAT1 and plasmid constructs

Specific primers for amplifying the salmon STAT1 gene were made based on the rainbow trout (Oncorhynchus mykiss) STAT1 sequence with the GenBank accession number U60331. The primers are listed in Table 1. The primers were Gateway compatible allowing the PCR fragment to be inserted into pENTR/D-TOPO vector (Invitrogen). A 2.3 kB fragment was amplified by *Pfu *DNA polymerase (Stratagene) using mixed cDNA from salmon ovary and HK obtained as described earlier [79]. Constructs were verified by DNA sequencing using the BigDye chemistry and a 3100 Gene Analyzer (Applied Biosciences). For transfection in cells, inserts were further transferred to the Gateway compatible eukaryotic expression vectors pDEST12.2 (Invitrogen), pDEST-GFP or pDEST-Myc (both provided by Dr. T. Lamark, University of Tromsø) by Gateway recombination using LR clonase II enzyme mix (Invitrogen) following manufacturer's instructions. For yeast two-hybrid analysis modified Clontech vectors (pGADT7 and pGBKT7) were used. The vectors (kindly provided by Dr. O. M. Seternes, University of Tromsø) were made Gateway compatible by insertion of the Gateway polylinker region as described [80] and are named pDESTGal4_AD _and pDESTGal4_DBD _respectively. Recombination into pGal4_DBD _required an intermediate cloning step into the pDONR207 vector (Invitrogen). Control plasmids pTD1-1, pGBKT7-53 and pGBKT7-Lam were purchased from Clontech.

### Phylogenetic analyses

Alignment of different STAT1 protein sequences from Atlantic salmon (ssSTAT1a, EU016199 and BT045567), Rainbow trout (U60331), Snakehead (EF079868), Green pufferfish (AF307105), Japanese flounder (EF491182), human (NM_007315), rat (NM_032612), African clawed frog (Xenopus, AY101602), Zebrafish (NM131480), Crucian carp (AY242386), mouse (NP_033309), pig (NP_998934), cow (NP_001071368), and chicken (NP_001012932). In addition other STATs (STAT2, STAT3, STAT4, STAT5 and STAT6 from some of these species were included in the analysis done by BioEdit and ClustalW version 1.81. A phylogenetic tree of STAT1 proteins was constructed using the neighbor-joining algorithm in clustalW. Bootstrap values were set to 1000.

### Real-time RT-PCR quantification

Total RNA was extracted from head kidney leukocytes using RNeasy^® ^Mini Kit (Qiagen) or from salmon organs using TRIzol^® ^(Invitrogen). RNA (300 ng in a 20 μl reaction) was reverse transcribed using TaqMan^® ^Reverse Transcription Reagents (Applied Biosystems). A volume of 2 μl of cDNA (6.25 ng of reverse-transcribed RNA) per 25 μl PCR reaction was used with primers for ssSTAT1 (ssSTAT1fw and ssSTAT1rev, see table 1) together with an ssSTAT1 probe (5'-6FAM-ACCACCAAGGAATGTTC-3'). While 6.25 pg of reverse-transcribed RNA per 25 μl PCR reaction was used to estimate 18S rRNA levels. The expression of mRNA was measured in an ABI Prism 7500 FAST Cycler (Applied Biosystems) using custom TaqMan^® ^assays designed by Applied Biosystems and FAST PCR mastermix (Applied Biosystems). The amplification profile was 95°C for 20 s followed by 40 cycles of 95°C for 3 s and 60°C for 30 s. All cDNA samples were performed in triplicates. The expression was normalized against EF1AB and presented as relative expression compared to the non-treated control sample. Relative expression was calculated using the Pfaffl's mathematical model [81]. The expression profiles from non-treated control fish organ samples were presented as expression of STAT1/EF1AB.

### Two-hybrid analysis

Both rich and selective yeast growth media were made from commercially available powders (Clontech). Yeast cells were grown at 30°C for 2 - 4 days. Plasmid constructs based on the pGal4_DBD _vector or the pGal4_AD _vector were transformed using Frozen-EZ Yeast Transformation II kit (Zymo Research) into competent yeast cells of strains *S. cerevisiae *Y187 (MATα) or PJ69-2A (MATa) by selecting for growth on medium lacking leucin or tryptophan, respectively. At least ten of each transformants carrying STAT1 were mated to each other. Ten diploid yeast cells from the mating were plated and scored for growth on a triple drop out medium (TDO) lacking leucin, tryptophan and histidine or a quadruple drop out medium (QDO) lacking leucin, tryptophan, histidine and adenine. Growth on TDO plates indicates a weak interaction, whereas growth on QDO plates indicates a stronger interaction. SV40 T-antigen (pTD1-1) and p53 (pGBKT7-53) served as positive controls, LaminC (pGBKT7-Lam) and empty vectors as negative controls.

### Transfection

For transfection, HEK-293, CHSE-214 or TO cells were seeded into 24-well plates with a density of 2 × 10^5 ^cells/well for HEK293 and CHSE-214, and 1 × 10^5 ^cells/well for TO, while in 6-well plates the densities were 1 × 10^6 ^or 5 × 10^5 ^cells/well, respectively. The cells were transfected the next day at 80 - 90% confluence. Transfection of the HEK-293 and CHSE-214 cells was performed by using the Lipofectamine 2000 (Invitrogen) transfection reagent according to the manufacturer's protocol. For each well, a total of 0.8 μg of plasmid DNA was incubated with 2 μl Lipofectamine 2000 in 100 μl serum-free EMEM for 20 min at room temperature before added to the cells. Three hours post transfection, FBS was added to a total concentration of 2%. For transfection of the TO cells the transfection reagent FuGENE HD (Roche Applied Science) was used according to the manufacturer's protocol. A total of 0.6 μg of plasmid DNA was mixed with 1.25 μl FuGENE HD in 50 μl EMEM and incubated 15 min before added to the cells with medium containing 2% FBS.

### Luciferase assay

TO cells transiently transfected with a Mx-promoter-luciferase construct [82] were lysed in 50 μl 1× passive lysis buffer (PLB), from the Dual-Luciferase Reporter Assay System (Promega). 100 μl of luciferase assay buffer II (LAR II) was predispensed in a luminometer plate (96 wells) and 20 μl of the lysate added (according to the manufacturer's protocol). The firefly luciferase activity [83] was measured in a Luminoscan RT luminometer (Labsystems OY), before 100 μl of Stop&Glo reagent was added and Renilla luciferase activity recorded for estimation of transfection efficiency (LAF/LAR). All samples for the luciferase assay were set up in triplicate and the results given as relative light units (RLU).

### Nuclear extraction

NE-PER Nuclear and Cytoplasmic Extraction Reagents (Thermo scientific) was used to extract nuclear and cytoplasmic proteins from 2 × 10^6 ^TO cells pr. reaction according to the manufacturer's protocol.

### Gel electrophoresis, Western blotting and antibodies

Cells were lysed in 50 μl sodium dodecyl sulfate (SDS) sample buffer (160 mM Tris-HCl [pH 6.8], 10% β-mercaptoethanol, 2% SDS, 20% glycerol, 0.1% bromophenol blue), transferred from the culture well into a microcentrifuge tube and boiled for 5 min. Then, typically 15-20 μl of the samples were loaded in each well of a precast 4-12% gradient NuPAGE Novex Bis-Tris gel and subjected to SDS-polyacrylamide gel electrophoresis (SDS-PAGE) with MES buffer and Western blotting using the Invitrogen NuPAGE system. Gel electrophoresis, blotting, blocking and antibody incubation were performed as described by the manufacturer. A polyclonal antibody against STAT1 (α-STAT1) was custom made from a C-terminal peptide retrieved from the salmon STAT1 sequence: RSVAPVFQCWTGPKE. The peptide was cross-linked by BSA to glutaraldehyde and two rabbits were injected with antigen every 14 days. The terminal bleed of the rabbits took place after day 68 (5 injections). The antiserum was antigen-purified and reactivity against the peptide was confirmed by dot blot analysis against the peptide. The specificity of the antiserum was checked by transfection and expression of a STAT1-GFP fusion construct in different cell-types and the results are presented as supplementary data (Additional file 3, supplemental Figure S2). A dilution of 1:2000 of the anti-STAT1 antibody (α-STAT1) was found to be appropriate for Western blotting. A polyclonal Mx antibody (α-Mx, 1:1000 dilution) [84] was applied as primary antibody for detection of Mx protein, and a GFP antibody (1:10000 dilution) (Abcam) for detection of GFP-tagged proteins. An actin antibody (1:1000 dilution) produced in rabbit (Sigma) was used as loading control in most Western blots and an antibody directed towards cod Cathepsin D (1:2000 dilution) [42] was used as a cytoplasmic marker. Goat anti-rabbit-Horseradish Peroxidase (HRP) antibody or goat anti-mouse-HRP antibody (Santa Cruz Biotechnology) diluted 1:25000 were used as secondary antibodies. Detection was performed by using SuperSignal West Pico chemiluminescent substrate (Pierce Biotechnology Inc.). Stripping of the membranes was performed in 0.2 M NaOH for 10 min followed by washing, blocking and new antibody incubation.

### Immunoprecipitation (IP) of STAT1

TO cells or Atlantic salmon spleen and HK leukocytes were stimulated with IFN-a1 or IFNγ for 1 and 3 h before washed two times with ice-cold PBS and harvested in buffer A (20 mM Tris-acetate, pH 7.0; 0.27 M sucrose; 1 mM EDTA; 1 mM EGTA; 1 mM orthovanadate; 10 mM β-glycerophosphate; 50 mM sodium fluoride; 5 mM sodium pyrophosphate; 1% [vol/vol] Triton X-100; 0.1% [vol/vol] 2-mercaptoethanol) and 'Complete' protease inhibitor cocktail (one tablet/50 ml, Roche). Lysates were cleared by centrifugation at 4°C for 15 min at 18000 × g. Lysates were then subjected to IP by incubating for 1 h at 4°C with αSTAT1 (1:100), before addition of 10 μl protein A-agarose (50% slurry pre-equilibrated in buffer A) and incubation at 4°C for 1 h. The immunoprecipitated material was washed four times in ice-cold buffer A with 0.5 M NaCl and resuspended in 40 μl 2× LDS-sample buffer. STAT1 was detected by the tyrosine phospho-specific antibody 4G10 Anti-Phosphotyrosine (Millipore) after SDS-PAGE and Western blotting.

### Co-IP analyses

HEK-293 cells co-transfected with the eukaryotic expression vectors pEXP12.2-STAT1 and pEXP-GFP-STAT1 or pEXP12.2-STAT1 and pEXP-GFP were washed two times with ice-cold phosphate-buffered saline (PBS) and harvested in HA-lysis buffer (50 mM Tris-HCl [pH 7.5], 150 mM NaCl, 2 mM EDTA, 1 mM EGTA, 1% Triton X-100 ) with a protease inhibitor cocktail added (Complete EDTA-free, Roche). Cell lysates were incubated on ice for 15 min and cleared by centrifugation for 15 min at 18000 × g in a microcentrifuge. Lysates were then subjected to IP with either α-STAT1 or α-GFP together with pre-blocked Protein A/G PLUS-Agarose beads (Santa Cruz biotechnology). The agarose beads were then washed four times with HA-lysis buffer, and all traces of buffer removed with a pipette tip before elution in 50 μl 2× SDS sample buffer. Eluted proteins were subjected to SDS-PAGE and visualized by Western blotting and antibody detection.

### Immunofluorescence microscopy

To examine localization of STAT1 in cells, primary cells from salmon HK were seeded on 14 mm coverslips. Non-adherent cells were washed away 1 day after seeding, and adherent monocyte/macrophages were stimulated with recombinant IFNs as described above. The cells were rinsed with 1× PBS (phosphate-buffered saline), before fixed using 4% paraformaldehyde for 20 min at room temperature. Cells were washed three times with PBS, and permeabilized with 0.3% Triton X-100 for 15 min, before blocked for 30 min with 7.5% FBS in PBS. The cells were then incubated with the primary antibody (α-STAT1) at a 1:500 dilution in PBS 7.5% FBS for 1 h washed three times with PBS and incubated with secondary antibodies conjugated to Alexa Fluor 546 at a 1:2000 dilution (Invitrogen) for 45 min away from light. Cells were washed and stained with DAPI (4',6'-diamidino-2-phenylindole, 1:300, Invitrogen) and mounted on slides with an antifade mounting medium. Confocal laser scanning microscopy was performed using a Leica TCS SP5 confocal microscope with LAS AF software.

## Authors' contributions

AS carried out or assisted with all of the experiments for this study, participated in its design and data-analyses and drafted the manuscript. TH designed and carried out the phosphorylation studies and assisted in drafting the manuscript. C-YS assisted in cloning and sequencing the ssSTAT1a gene and performed the Y2H interaction studies. HLT performed the qPCR analyses and assisted in providing primary leukocytes. JBJ conceived of and coordinated this study, assisted in data analyses and edited the manuscript. All authors read and approved the final manuscript.

## Supplementary Material

Additional file 1 **Supplemental Table S1**. Percent amino acids sequence identities (top right triangle) and similarities (bottom left triangle) of STAT1 proteins. The accession numbers for the STAT1 from each species are given in parentheses. Click here for file

Additional file 2 **Supplemental Figure S1. Over-expression of ssSTAT1a in TO cells does not increase the induction of the Mx-promoter significantly over IFN-induced levels**. TO cells were transiently co-transfected with a Mx promoter-luciferase reporter construct and ssSTAT1a or an empty expression vector. Forty-eight hours after transfection, the cells were left untreated or stimulated with IFN-a1 (10 U/mL) and IFNγ (200 ng/ml) for 4, 6 and 8 h prior to measurement of luciferase activity. The relative luciferase activity was normalized against Renilla luciferase activity. Each bar represents the average of triplicate determinations from a representative experiment that was repeated two times with similar results. Error bars indicate standard deviation. Click here for file

Additional file 3 **Supplemental Figure S2. The specificity of the STAT1 peptide antibody **was checked by transfection and expression of a GFP-ssSTAT1a fusion construct in different cell-types followed by SDS-PAGE and Western blotting. GFP-ssSTAT1a was expressed and recognized by the STAT1 antibody and a GFP antibody in HEK-293 cells and CHSE-214 cells, whereas in TO cells the level of transfected GFP-ssSTAT1a was undetectable while endogenous expression of STAT1 was detected in these cells. Actin was used as a loading control. Arrowheads indicate unspecific bands cross-reacting to the GFP antibody. Click here for file
