# Supplementary material for: Structural and functional studies of STAT1 from Atlantic salmon (Salmo salar)
Source: BMC Immunol. 2010 Mar 30;11:17. doi: 10.1186/1471-2172-11-17 (PMC2855521; doi:10.1186/1471-2172-11-17)
Supplement: Additional file 1 — Supplemental Table S1. Percent amino acids sequence identities (top right triangle) and similarities (bottom left triangle) of STAT1 proteins. The accession numbers for the STAT1 from each species are given in parentheses. [file 1471-2172-11-17-S1.PDF]

Supplemental Table 1. Percent amino acids sequence identities (top right triangle) and similarities (bottom left triangle) of STAT1 proteins. The accession numbers for the STAT1 from each species are given in parentheses.

	ssSTAT1a	Atlantic salmon (EU016199)	Atlantic salmon (BT045567)	Rainbow trout (U60331)	Snakehead (EF079868)	Green pufferfish (AF307105)	Japanese flounder (EF491182)	Human (NM_007315)	Norway rat (NM_032612)	African clawed frog (AY101602)	Zebrafish (NM_131480)	Crucian carp (AY242386)
ssSTAT1a		96.3	98.3	97.6	80.1	76.4	77.9	66.1	64.7	65.8	63.3	58.4
Atlantic salmon (EU016199)	96.7		94.6	94.9	79.9	76.2	78.0	66.3	65.1	65.7	63.6	59.5
Atlantic salmon (BT045567)	98.3	95.1		95.9	79.1	75.8	76.8	65.4	64.1	65.2	62.6	57.4
Rainbow trout (U60331)	98.8	96.4	97.1		80.1	76.5	78.1	66.8	65.4	66.2	63.2	58.6
Snakehead (EF079868)	90.4	90.0	89.3	90.5		86.1	88.4	66.5	66.0	65.7	62.8	58.3
Green pufferfish (AF307105)	87.5	86.9	87.1	87.2	92.9		83.1	65.2	64.8	63.8	60.8	57.1
Japanese flounder (EF491182)	89.2	89.0	88.0	89.5	94.0	92.2		66.0	65.7	63.8	63.0	57.9
Human (NM_007315)	82.4	82.3	81.7	82.8	83.9	81.9	82.9		93.6	79.0	59.9	58.4
Norway rat (NM_032612)	81.5	81.6	80.8	81.8	83.2	81.4	82.8	97.2		78.2	59.8	59.1
African clawed frog (AY101602)	82.0	82.0	81.3	82.6	83.1	81.2	82.2	90.6	89.5		59.8	57.1
Zebrafish (NM_131480)	79.9	80.1	79.1	80.2	79.8	79.8	80.7	79.2	78.9	79.1		52.8
Crucian carp (AY242386)	75.7	77.1	74.4	75.9	76.2	75.2	76.5	76.8	76.5	76.1	71.5	
