# Supplementary figures and images for: Structural and functional studies of STAT1 from Atlantic salmon (Salmo salar)
Source: BMC Immunol. 2010 Mar 30;11:17. doi: 10.1186/1471-2172-11-17 (PMC2855521; doi:10.1186/1471-2172-11-17)

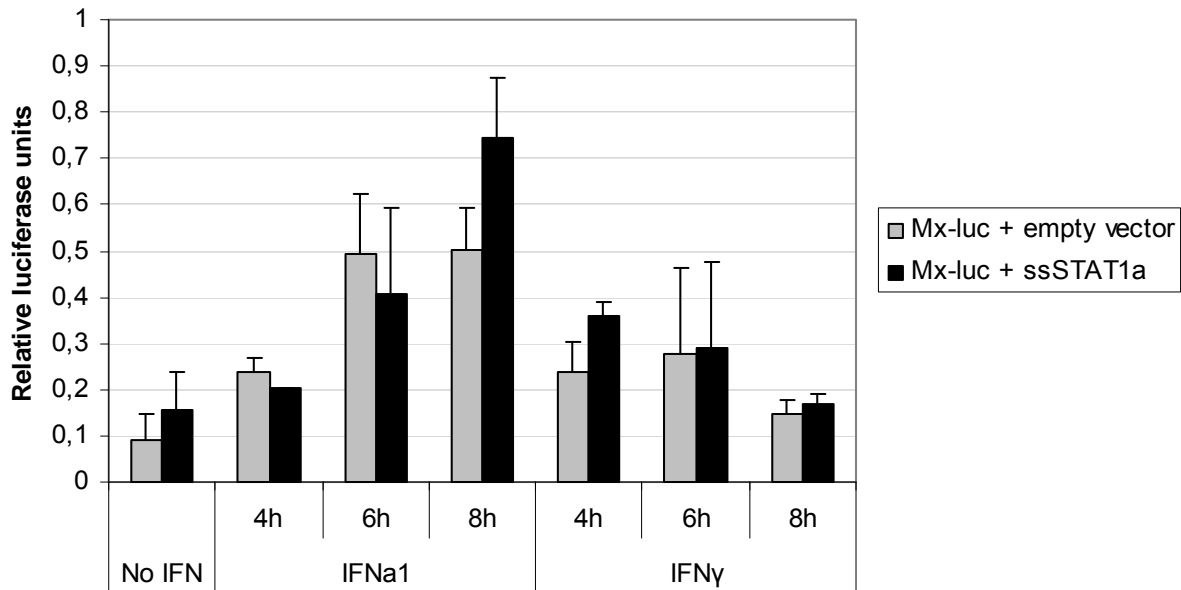

Supplement: Additional file 2 — Supplemental Figure S1. Over-expression of ssSTAT1a in TO cells does not increase the induction of the Mx-promoter significantly over IFN-induced levels. TO cells were transiently co-transfected with a Mx promoter-luciferase reporter construct and ssSTAT1a or an empty expression vector. Forty-eight hours after transfection, the cells were left untreated or stimulated with IFN-a1 (10 U/mL) and IFNγ (200 ng/ml) for 4, 6 and 8 h prior to measurement of luciferase activity. The relative luciferase activity was normalized against Renilla luciferase activity. Each bar represents the average of triplicate determinations from a representative experiment that was repeated two times with similar results. Error bars indicate standard deviation. [file 1471-2172-11-17-S2.PDF]

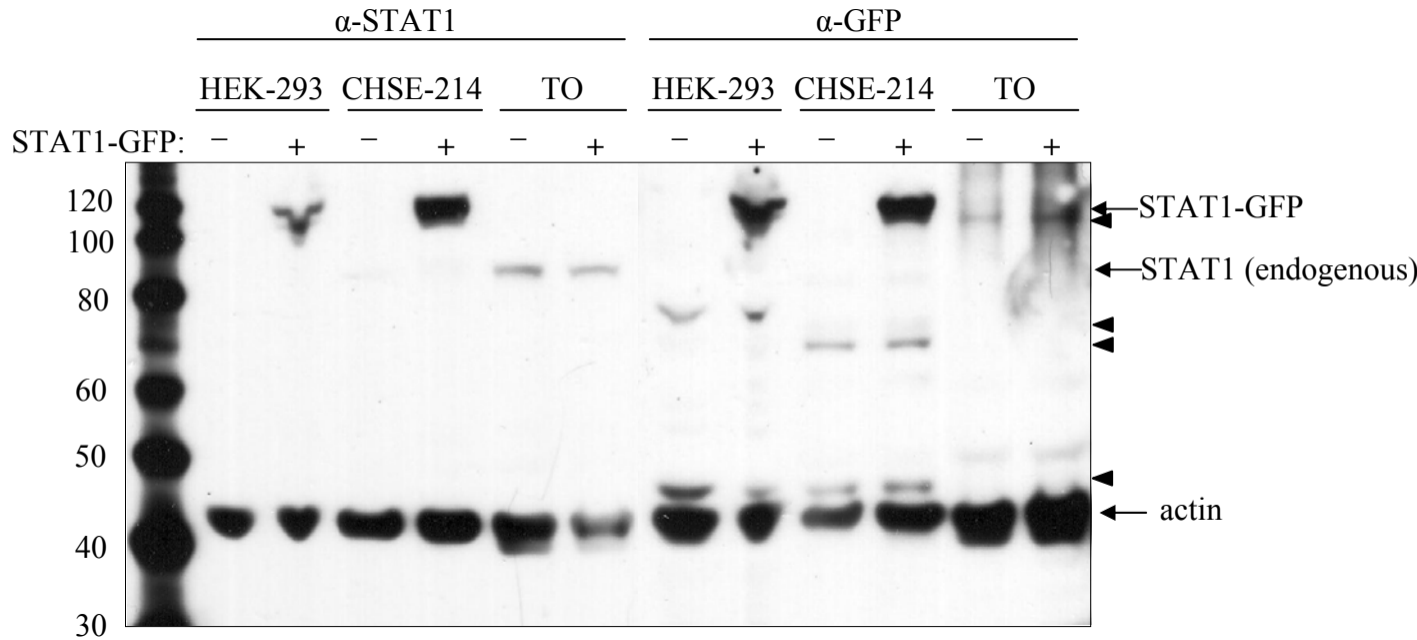

Supplement: Additional file 3 — Supplemental Figure S2. The specificity of the STAT1 peptide antibody was checked by transfection and expression of a GFP-ssSTAT1a fusion construct in different cell-types followed by SDS-PAGE and Western blotting. GFP-ssSTAT1a was expressed and recognized by the STAT1 antibody and a GFP antibody in HEK-293 cells and CHSE-214 cells, whereas in TO cells the level of transfected GFP-ssSTAT1a was undetectable while endogenous expression of STAT1 was detected in these cells. Actin was used as a loading control. Arrowheads indicate unspecific bands cross-reacting to the GFP antibody. [file 1471-2172-11-17-S3.PDF]
